# Supplementary material for: Efficacy of aspirin, clopidogrel, and ticlopidine in stroke prevention: A population-based case-cohort study in Taiwan
Source: PLoS One. 2020 Dec 28;15(12):e0242466. doi: 10.1371/journal.pone.0242466 (PMC7769270; doi:10.1371/journal.pone.0242466)
Supplement: S1 Table — (DOCX) [file pone.0242466.s001.docx]

S1 Table: Condition of antiplatelet use

|  | Number | % |
| --- | --- | --- |
| Aspirin only | 11284 | 66.9% |
| Aspirin-Combine(≥2 agents) | 332 | 1.97% |
| Licodin only | 444 | 2.63% |
| Licodin-Aspirin | 1166 | 6.91% |
| Licodin-Aspirin-Combine(≥2 agents) | 137 | 0.81% |
| Licodin-Combine(≥2 agents) | 20 | 0.12% |
| Licodin-Plavix | 124 | 0.74% |
| Licodin-Plavix-Aspirin | 326 | 1.93% |
| Licodin-Plavix-Aspirin-Combine(≥2 agents) | 44 | 0.26% |
| Licodin-Plavix-Combine(≥2 agents) | 11 | 0.07% |
| Plavix only | 834 | 4.94% |
| Plavix-Aspirin | 1738 | 10.3% |
| Plavix-Aspirin-Combine(≥2 agents) | 296 | 1.76% |
| Plavix-Combine(≥2 agents) | 61 | 0.36% |
| Combine(≥2 agents) only | 49 | 0.29% |

Licodin: ticlopidine 250 mg/day, Plavix: clopidogrel 75 mg/day

Aspirin: aspirin 100mg/day
