# Supplementary material for: Efficacy of aspirin, clopidogrel, and ticlopidine in stroke prevention: A population-based case-cohort study in Taiwan
Source: PLoS One. 2020 Dec 28;15(12):e0242466. doi: 10.1371/journal.pone.0242466 (PMC7769270; doi:10.1371/journal.pone.0242466)
Supplement: Attachment — Submitted filename: Response to reviewer 2.docx [file pone.0242466.s002.docx]

Response to reviewer 2

We thank reviewer for their valuable comments. Base on reviewer’s comments, we have made necessary revision.

1. The methods of data collection should be more detailed. How was the 1-million sample ‘randomly’ collected? Why chosen for 1-million sample instead of directly identifying patients from the NHIRD based on discharge diagnosis in a certain time span?

Revision: In the methods section, we have add the information about the methods of data collection. “The current study included the data were retrieved from the “Longitudinal Health Insurance Database (LHID 2005) from a random sample of 1 million individuals within the NHIRD, with linked longitudinal data available from 2000 to 2010. The LHID 2005 contains complete medical claims and registration for a random sample of one million individuals within the NHIRD. The randomized data (LHID 2005) are overall representative of all beneficiaries as no significant difference in the sex, age and premium rate between individuals in the LHID 2005 and in the original NHIRD data sets.”

2. Of the patients identified with primary diagnosis ischemic stroke (n=19543) >50% were excluded. As this is a large number excluded from the population of interest (in a cohort study), I would like to gain more insight in the exclusion criteria.

Revision: In the methods section, we add the exclusion criteria. “We excluded the	patients who did not use of one of the three antiplatelets drugs for more than	14 days within the first month after the stroke and the patients not continued	use of the antiplatelet drugs were not included in the study.”

3. To be able to interpret the role of different antithrombotic agents, not only thrombotic risks should be reported, but also bleeding risks. Is there any information available on bleeding complications among patients on aspirin vs ticlopidine vs clopidogrel therapy?

Revision: In the results and discussion section, we add the information about hemorrhage stroke and neutropenia.

In results section: “The hemorrhagic stroke rates were no significant difference among 3 groups (p=0.85), 1.40% (29/2068), 1.48% (3/203), and 0.95% (3/314) in the aspirin, ticlopidine, and clopidogrel groups, respectively. Neutropenia was found in 0.29% (6/2068) of patients receiving aspirin, no neutropenia was found in patients receiving ticlopidine and clopidogrel.”

In discussion section: “Hematologic abnormalities including neutropenia and thrombocytopenia attribute to ticlopidine had been reported. In the studies which patient use ticlopidine 500mg/day, the incidence of neutropenia was between 0.6% and 3.4%.^27-30^. The incidence of neutropenia in patients use ticlopidine 250mg/day was between 0.29% and 0.37%.^31, 32^ The incidence of neutropenia seems related to the dose of antiplatelet.

The incidence of neutropenia in the patients use aspirin 650 mg/day is 2.2%.^30^ Lower ticlopidine dose can reduce the risk of neutropenia. In the study, neutropenia was not found in patients receiving ticlopidine and clopidogrel. The result suspect related to our patients receiving low dose of ticlopidine (250mg/day). Our study found the risk of intracranial hemorrhage is no significant difference among the patients receiving aspirin, ticlopidine and clopidogrel. The result is the same as previous studies that the hemorrhage stroke rate was no significant difference between aspirin and clopidogrel^1,13^ and between aspirin and ticlopidine.^23^

4. Ticlopidine was found to be non-inferior to aspirin. However, ticlopidine use is world-wide mainly limited due to its serious side effect such as neutropenia and TTP. Although data on neutropenia and thrombocytopenia were not available in the NHIRD, I believe the prevalence of these ticlopidine side effects among Asian population should be highlighted more in the discussion to put results in better perspective.

Revision: Neutropenia and hemorrhage adverse effect was reported and discussion as issue 3.

5. • The manuscript reports that, based on the time-dependent Cox proportional hazards model analysis, length of stay was associated with increased risk of recurrent stroke. However, the crude HR for length of stay >9d was 1.03 (0.54-1.98).

Revision: We have deleted length of stay.

6. The manuscript reports that after adjusting for risk factors, length of stay and thrombocytopenia were associated with increased risk of recurrent stroke. However, the adjusted HR for length of stay >9d was 1.01 (0.52-1.99) and for thrombocytopenia was 3.16 (0.85-11.76).

Revision: We have deleted length of stay and thrombocytopenia.

7. Add reference in the last paragraph of the Discussion stating: “…Altogether, these results suggest that a ticlopidine dose of 500mg/day may not be necessary for the prevention of recurrent stroke and that the adverse event of severe neutropenia might be related to the high ticlopidine dose.”

Revision: In discussion section, we have add the reference about the relationship between ticlopidine dose and neutropenia or thrombocytopenia. “Hematologic abnormalities including neutropenia and thrombocytopenia attribute to ticlopidine had been reported. In the studies which patient receiving ticlopidine 500mg/day, the incidence of neutropenia was between 0.6% and 3.4%.^27-30^. The incidence of neutropenia in patients use ticlopidine 250mg/day was between 0.29% and 0.37%.^31, 32^ The incidence of neutropenia seems related to the dose of antiplatelet.

The incidence of neutropenia in the patients use aspirin 650 mg/day is 2.2%.^30^ Lower ticlopidine dose can reduce the risk of neutropenia. In the study, neutropenia was not found in patients receiving ticlopidine and clopidogrel. The result suspect related to our patients receiving low dose of ticlopidine (250mg/day). Our study found the risk of intracranial hemorrhage is no significant difference among the patients receiving aspirin, ticlopidine and clopidogrel. The result is the same as previous studies that the hemorrhage stroke rate was no significant difference between aspirin and clopidogrel^1,13^ and between aspirin and ticlopidine.^23^
