# Supplementary material for: Efficacy of aspirin, clopidogrel, and ticlopidine in stroke prevention: A population-based case-cohort study in Taiwan
Source: PLoS One. 2020 Dec 28;15(12):e0242466. doi: 10.1371/journal.pone.0242466 (PMC7769270; doi:10.1371/journal.pone.0242466)
Supplement: Attachment — Submitted filename: Response to reviewer 3.docx [file pone.0242466.s004.docx]

Response to reviewer 2

Thanks reviewer’s comments, based on reviewer’s comments we have made necessary change.

1. Of patients identified with primary diagnosis ischemic stroke (n=19543) >50% were excluded. The authors report in the revised version exclusion of the patients who did not use one of the three antiplatelet drugs for more than 14 days within the first month after the stroke and the patients not continued use of the antiplatelet drugs. I recommend the authors to explain this latter exclusion criteria, be more specific: discontinuation after what period of treatment. Discontinuation of antiplatelet drugs during treatment can also be due to bleeding or ischemic complications. In that case exclusion of these patients resulted in selection bias with possible influence of study results.

Response: In methods “Study subjects and definitions” section, Page 8 to page 9, we add a sentence “We believe the patients who did not regularly use antiplatelet drugs potentially poor medical compliance. To reduce bias, we excluded the patients who did not use antiplatelet drugs for more than 14 days within the first month after the stroke and the patients who had not use antiplatelet drugs for more than 90 consecutive days .”

In figure 1, we add an exclusion criteria, “(7). Patients who have not use antiplatelet drugs for more than 90 consecutive days (n=1604).”

2. Figure 1 reports 8,806 patients screened for inclusion. Based on the exclusion criteria, 8806-2085=6721 patients should be included for propensity score matching (2085 = sum of criteria 1-6 in figure). Author report 5117, thus 6721 – 5117 = 1604 patients were excluded currently for unknown reason. Please correct the numbers or report the reason of exclusion of these 1604 patients.

Response: In figure 1. We add an exclusion criteria. “(7). Patients who had not use antiplatelet drugs for more than 90 consecutive days (n=1604).”
