# Supplementary material for: Efficacy of aspirin, clopidogrel, and ticlopidine in stroke prevention: A population-based case-cohort study in Taiwan
Source: PLoS One. 2020 Dec 28;15(12):e0242466. doi: 10.1371/journal.pone.0242466 (PMC7769270; doi:10.1371/journal.pone.0242466)
Supplement: Attachment — Submitted filename: Response to reviewer 2.PONE4.docx [file pone.0242466.s005.docx]

Response to reviewer 2

We thanks reviewer’s valuable comments. We very concern selection bias, because it may influence the result, so we excluded the patients who potential poor medical compliance. However, it may also excluded the patients who discontinue antiplatelet due to major adverse effect of antiplatelet. It is the limitation of the retrospective study from the data base. We add a paragraph in limitation section. Two issue was point out by reviewer, we have made necessary revision.

1. Is the manuscript presented in an intelligible fashion and written in standard English?

Response: The English have been proofreading and editing by Enago-Taiwan

(enago.tw).

2. Thank you for considering my comments. However, I still have concern regarding possible selection bias in this study.

You describe that patients did not use APT for more than >90 consecutive days due to poor medical compliance and therefore you excluded these patients form this study to reduce bias. However, it is not known whether this hypothesis is correct. It is likely that patients did not use APT for >90 days due to bleeding complications. And if poor medical compliance played a role, it could also be the result of nuisance bleeding. I would have suggested to include also patients not that did not use APT for more than >90 consecutive days in the primary analysis and afterwards, exclude this group in a sensitivity analysis.

However, as the study is now, I believe that there is a possible relevant selection bias which at least should be mentioned as a limitation in the discussion.

Response:

In the limitation section, we add a limitation “Fifth, we excluded patients who did not regularly use antiplatelet drugs, which may also exclude patients who discontinued antiplatelet medications due to an adverse effect. These exclusions may underestimate the risk of adverse effects of antiplatelet medications. However, in Taiwan, when adverse effects occur, most patients will come to the hospital for help, and physicians will change the drug but will not discontinue medications.”
